# Acceptability of the social uses of the COVID-19 screening test among women in southern Benin

**DOI:** 10.4102/jphia.v16i1.810

**Published:** 2025-02-07

**Authors:** Mingnimon A. Affo

**Affiliations:** 1 Centre for Training and Research in Population, University of Abomey-Calavi, Abomey-Calavi, Benin; 2 Multidisciplinary Doctoral School, Faculty of Human and Social Sciences, University of Abomey-Calavi, Abomey-Calavi, Benin; 3 Population Health and Development Research Group, University of Abomey-Calavi, Abomey-Calavi, Benin

**Keywords:** acceptability, COVID-19, women, informal sector, Benin

## Abstract

**Background:**

Screening tests are some of the essential measures in the fight against all diseases with epidemic potential. The refusal to use it is the major challenge that hinders this fight.

**Aim:**

This article aims to highlight the factors for the rejection of the COVID-19 screening test among women in the informal sector in Benin.

**Setting:**

The data were collected in southern Benin.

**Methods:**

A cross-sectional approach was used to collect data in two areas (intervention area and buffer zone). The sample was drawn using a two-stage random sampling design. In the first stage, primary sampling units or clusters or villages or neighbourhoods were drawn, and in the second stage, 40 households were selected by primary sampling units. Overall, 2500 households per area in which about 2500 women aged 15–64 years were interviewed. Descriptive and explanatory analyses were carried out.

**Results:**

The results show that a strong majority (84.2%) of respondents showed aversion to the COVID-19 screening test. Individual factors (age, level of education, religion) and contextual factors (sectors and types of activities of the respondents) are the main reasons behind this refusal.

**Conclusion:**

Insufficient consideration of local contexts around health emergencies, infodemia and social inequalities in health have contributed to aversion to the COVID-19 screening test.

**Contribution:**

The results call on public authorities to support a constant improvement of knowledge on COVID-19 taking into account local approaches to facilitate the adherence of populations to the screening test.

## Introduction

The World Health Organization (WHO) declared severe acute respiratory syndrome coronavirus-2 (SARS-CoV-2) or coronavirus disease 2019 (COVID-19) a public health emergency of international concern and considered it a pandemic in January 2020. To this end, it has put in place a set of online technical guidance as well as advice for all countries on how to detect, screen and manage potential cases, based on what is known about the virus.^1^ Large-scale screening by reverse transcription polymerase chain reaction (RT-PCR) virological test has been proposed to the population as part of the response measures. These tests make it possible to detect cases of infected people asymptomatic or pre-symptomatic and may go unnoticed, including vaccinated populations.^2^ Thus, they allowed early treatment and reduced exposure to COVID-19 and ensured the safety of the population.^3^

One of the challenges of screening in Africa is developing efficient mechanisms to get populations to accept this test at a time when the social representations of this disease are causing many controversies, ranging from the numinous to the conspiracy theory underpinned by the Euro-Western ‘coloniality’ of knowledge and practices.^4,5^ Indeed, the advent of COVID-19 has been largely perceived in Africa as a creation of the West and testing, including vaccination, considered as a means of controlling the African population.^6^ According to some authors, moving away from ‘coloniality’ could lead to a move away from misleading universalism or ‘Western universals’ to position oneself in the field of knowledge that reflects the plural nature of the world.^7,8^

Research works have focussed on social engagement in the fight against the COVID-19 pandemic through the analysis of the acceptability of the screening test, as it were for vaccination programmes. On these aspects, case studies have investigated low-resource contexts, particularly in sub-Saharan Africa. The investigations carried out in Brazzaville (Democratic Republic of Congo) during the peak period of the disease^9^ suggest that local populations are distrustful of, or rather averse to, the mechanisms used to combat COVID-19. It emerged that the factors associated with the refusal of voluntary testing were mainly fear of the results and low perception of the severity of the disease. The results also indicated that while acceptance of voluntary COVID-19 testing increased with the perception of disease severity, a low level of knowledge of transmission channels or disease severity was associated with its refusal. It follows that overall, non-acceptance of voluntary COVID-19 testing was mainly related to fear of the test result or denial of the disease.^9^

The speed of human-to-human transmission of COVID-19 has led the WHO to declare the disease a public health emergency of international concern, and a pandemic. Therefore, screening appears to be efficient strategy for prevention and control. In Benin, the first case of COVID-19 was diagnosed on 16 March 2020 at the Laboratory of Viral Hemorrhagic Fevers in Cotonou.^10^ At the beginning of the pandemic, the country, like many other African countries, could not carry out the tests that met its needs. But very quickly, after a few months, African governments increased their diagnostic capacities, which were essential to understand the scale and dynamics of infections as well as adjusting public health measures in response to the virus.

Benin has gone from a single laboratory in Cotonou to 13 laboratories capable of carrying out tests, at least one in each of its 12 departments. Several control strategies have been deployed. Different types of tests have been implemented to identify, break the avenues of transmission of the virus and control the evolution of the pandemic. The main tests deployed were virological (RT-PCR), rapid antigen tests and serological tests. It should be recalled that a toll-free number was first created in April 2020 to inform and direct the population to the sorting and screening centres that have gradually been opened in all 77 municipalities of the country. Testing sites were set up at the airport and borders, and mobile testing teams also visited administrative institutions and businesses. We also noted the creation of a Cordon Sanitaire cutting off the south of the country (COSAN zone, considered as the gateway and area of concentration of the disease) from the hinterland in order to limit the spread of the disease.

Benin has thus been able to carry out more than 7900 tests per week, that is, more than 222 000 tests in total since the beginning of the pandemic thanks to the establishment of the screening network whose mission is to detect, alert and track down suspected cases of COVID-19 throughout the country.^11^ The government, keen to strengthen its diagnostic capacity, wanted to make the viral haemorrhagic fever laboratory a benchmark facility by forging a partnership with the Institut Pasteur in Dakar and the Institute of Virology at the Charité University Hospital in Berlin. This has made it possible to raise the level of detection of COVID-19 cases in Benin. Despite all the efforts made in the response to COVID-19 through screening tests from the beginning of the pandemic, Benin, as well as other countries in the sub-region, is still faced with the non-use or reluctance to take COVID-19 screening tests. A recent study of 5014 households with 4931 women aged 15–64 years in the informal sector in the south and centre of the country indicated that 84.2% had not been tested for COVID-19.^12^ The present analyses aim to study the explanatory factors for the non-use of COVID-19 testing among women in the non-formal sector in the south and centre of Benin.

Recent research works on acceptability have tended to focus on vaccination.^13^ Those specifically focussed on the acceptability of the screening test seem to be rather rare in sub-Saharan Africa. We assume that the acceptability of intervention in the community is underpinned by a complex rationality based on the collective beliefs and personal values of individuals.

The framework for analysing the acceptability of preventive measures enacted by public authorities also mobilises concerns about inequalities in health and well-being, the causes of which reveal, as emphasised by certain studies^14^, the complexity (through their cumulative and delayed effect over time) of the mechanisms by which social factors affect health (and vice versa) at different levels (individuals, communities, populations) and interacting with the other (individuals, communities, populations) and with other determinants of health (behaviour, psychology, environment, biology and structure of health systems). For the purposes of this study, the term ‘non-uptake’ is used to refer to any form of reluctance to undergo COVID-19 screening. It takes into account concepts such as non-acceptability, refusal or lack of recourse.

## Research methods and design

### Data

The data originated from the study of the effects of COVID-19 among women in the non-formal sector in the south and centre of Benin.^12^

The study targeted two areas (intervention area [Cordon Sanitaire zone {COSAN Zone}] and buffer zone [non Cordon Sanitaire zone {COSAN Zone}], in the Collines department and located 200 km from the COSAN Zone) with 2500 households per zone in which 2500 women aged 15–64 years and 150 men aged 15–64 years were interviewed. In total, for the two areas, a theoretical sample of 5000 households in which 5000 women aged 15–64 years and 2500 men aged 15–64 years are interviewed. The sampling frame consists of the file of villages or city districts established by the National Institute of Statistics and Economic Analysis (INSAE) during the 2013 General Census of Population and Housing (GCPH) of Benin. In general, in rural areas, the sampling unit is a village, while in urban areas, the sampling unit is a neighbourhood. The type of residence of a village or district is defined pertaining to the population size considered by the census. The sample was drawn using a two-stage random sampling design similar to that used in the Benin Demographic and Health Survey (EDSB-V) carried out in 2017–2018 by the National Institute of Statistics and Demography. In the first stage, primary sampling units or clusters or villages or neighbourhoods were drawn, and in the second stage, 40 households were selected by primary sampling units.

The study took place in eight municipalities selected randomly, four per area. In the COSAN zone, the selected municipalities are: Abomey-Calavi, Cotonou, Ouidah and Porto-Novo. In the non-COSAN zone, the municipalities of Save, Dassa-Zoume, Glazoue and Savalou have been selected. Anticipating that an average of 40 households will be targeted per cluster and adjusting the sample size upwards, to 2520 households, 63 clusters per survey intervention area (COSAN and non-COSAN) were formed. The distribution of the number of clusters per municipality within each investigation area (COSAN and non-COSAN) was carried out in proportion to the size of each municipality in terms of the number of clusters per municipality in the GCPH dated 2013 and then adjusted.

In each locality (village or city district) selected at the first stage, households were selected (drawn) according to the so-called ‘bottle’ technique. This random strategy makes it possible to reduce the potential selection biases that would be linked to the fact of letting the interviewer deliberately choose directions that suit him or her for one reason or another. Thus, the interviewer, after a guided tour of the locality (village or city district), stood in the centre. He spun a bottle on the floor or threw a pen in the air. Then, he moved in the direction indicated by the tip of the bottle or pen to the boundary of the locality (or at most 500 m from his starting point) counting (recorded in the count slip) all the households in which he visits. In his movement, he carefully marked (ticked) the eligible households (those in which women aged 15–64 years lived). Then, he calculates the sampling interval (no sampling) by dividing the number of eligible households enumerated by the expected sample size per village or neighbourhood (40 in this case). Table 1 shows the breakdown of the sample by study area.

**TABLE 1:** Allocation of the number of clusters and households by municipality and by project intervention area (COSAN and non-COSAN).

Intervention zone	Department	Commune	Number of households in the GCPH-2013	Number of clusters/village-neighbourhoods	Number of households
COSAN	Littoral	Cotonou	166 433	20	800
	Atlantique	Abomey-Calavi	145 510	18	720
		Ouidah	36 459	11	440
	Ouémé	Porto-Novo	60 368	14	560
Total COSAN			408 770	63	2520
Non-COSAN	Collines	Dassa-Zoumè	22 647	16	640
		Glazoué	22 333	16	640
		Savalou	28 001	19	760
		Savè	16 096	12	480
Total non-COSAN			89 077	63	2520
Together			497 847	126	5040

*Source*: UAC. Etude sur les effets de la COVID-19 chez les femmes du secteur non formel au Sud et Centre Bénin. University of Abomey-Calavi; 2023

COSAN, Cordon Sanitaire zone; GCPH, General Census of Population and Housing.

After obtaining the survey step (*p*), the researcher and the members of his team cut out small pieces of paper on which he wrote the numbers from 1 to *p*. He then grouped all the tickets thus cut into a jar, asked the chief of the locality or his representative (who served as a facilitator for the team of interviewers) to randomly choose one of the tickets that now represents the first sample household to be interviewed. After this cleaning, the team evolved in the same direction and counted a number of eligible households (inside houses and concessions) equivalent to the draw step. The latter is the second sample household to be interviewed. The team followed the draw step (repeated the operation) to determine the other sample households. Although the household is the secondary unit in this process, it is women aged 15–64 years who are the main target of the survey. One eligible woman is surveyed per household. In households where more than one woman is identified, the data collection application generates the respondent for the individual woman questionnaire after completing the list of household members. In addition, as mentioned earlier, men aged 15–64 years were interviewed in 20 of the 40 households in which eligible women were randomly selected and interviewed according to the same principle as that used for the selection of women. Table 1 shows the allocation of localities (neighbourhoods or villages) and households by project intervention area. The villages and city districts were randomly selected from the village register drawn up during the GCPH of 2013.

These analyses focussed on the sample of women. Two types of tools were used for this purpose for data collection. These are: (1) the household enumeration slip and (2) the questionnaires (household, woman). The enumeration slip named all households identified in the selected part (segment) of each cluster. The household questionnaire is administered to the heads of household or their representative (any other person who is at least 18 years of age and able to provide information about the household). The female questionnaire was administered to randomly selected women aged 15–64 years. Quantitative data were collected using tablets or smartphones. To this end, an input mask in Survey-CTO (Computer-Assisted Personal Interviewing [CAPI] Toolkit) has been developed for validated collection tools. The necessary checks to facilitate collection and minimise data entry errors are made and considered in the masks at the end of this survey. At the end of the collection, the data were compiled and audited to ensure the quality of the database before starting the analyses. Any information that could be used to identify a respondent is deleted before starting the analysis and before making the database accessible.

### Data analysis

Data were analysed using descriptive analysis techniques (univariate and bivariate) with the aid of the Statistical Package for Social Sciences (SPSS) software (IBM SPSS, Chicago, Illinois, United States). Proportions were calculated to determine the values of the indicators. The analysis was based on the examination of statistical tables reflecting the different objectives of the study. The significance threshold of the deviations is 5% (*p* < 0.05). Firstly, a bivariate analysis was carried out, consisting of the cross-referencing of each independent variable with the study variable, by relating them and establishing the level of this association using Chi-square. The dependent variable is the ‘non-use of the COVID-19 screening test’. It takes the code ‘1’ when the respondent refuses to take the COVID-19 screening test and ‘0’ otherwise. Based on the literature review and the data available in the study database, the independent variables used are: age, religion, marital status, level of education, place of residence, study area, main activity, field of activity and socio-professional category. Secondly, a simple binomial logistic regression was performed on the database to identify the factors influencing the phenomenon under study, using the non-use of COVID-19 screening as a dependent, qualitative and dichotomous variable. The overall Chi-square associated with the model is significant (*p* < 0.000), and the pseudo-*R*-square ranges from 0.023 to 0.040. As a prelude to the logistic regression, a collinearity analysis was carried out using the Chi-square test and Cramer’s V statistic, by crossing the explanatory variables in pairs. This analysis revealed that there were no confounding factors in the database. These different methods were chosen because of the qualitative nature of all the variables used in the study.

### Ethical considerations

Ethical approval to conduct this study was obtained from the Institute of Applied Biomedical Sciences Research Ethics Committee (No. 127) and the National Institute of Statistics and Demography (No. 26/2023/MEF/INStaD/DCSFM).

## Results

### Respondent profile

Table 2 shows that the respondents were relatively young and poorly educated. Four in ten (42.3%) were under 30 years old. Almost the same proportion (39.3%) were not educated. Quartz had a primary level education (25.7%) and about four in ten (35.0%) have a secondary level or higher level of education (see Table 2).

**TABLE 2:** Distribution (%) of respondents by selected socio-demographic characteristics.

Socio-demographic characteristics	%	Size
**Age group (years)**		
15–29	42.3	2085
30–49	42.9	2115
50 and older	14.8	731
**Religion**		
Vodoun	8.8	433
Muslim	13.2	649
Christian	74.9	3691
No religion	3.2	158
**Marital status**		
Single	14.8	731
In union	73.4	3620
Separated/divorced/widowed	11.8	580
**Level of education**		
None	39.3	1938
Primary	25.7	1268
Secondary or higher	35.0	1725
**Place of residence**		
Urban	41.7	2058
Rural	58.3	2873
**Study area**		
COSAN Zone	49.3	2429
Non COSAN Zone	50.7	2502
**Department**		
Littoral	15.9	785
Atlantique	22.4	1105
Ouémé	10.9	539
Collines	50.7	2502
**Main activity**		
Study/Apprenticeship	7.7	354
Home/unemployed	14.3	655
Work	78.0	3580
**Field of activity**		
Agriculture/animal husbandry	26.4	946
Handicraft	14.6	522
Commerce	53.0	1896
Service	6.0	216
**Industry**		
Informal	96.0	3438
Formula	4.0	142

**Total**	**100.0**	**4931**

COSAN, Cordon Sanitaire zone.

The majority of respondents lived in rural areas at the time of the survey (58.3% compared to 41.7% in urban areas). Half of them lived in the Cordon Sanitaire zone (COSAN) and the other half, in the control or buffer zone. More than seven in ten respondents (73.4%) were in unions. 14.8% of the respondents were single. The informal sector employed 96.0% of employed women. Nearly eight out of ten women (78.0%) were working at the time of the survey. More than half of women (53%) worked in commerce, particularly in the COSAN zone (71.5% compared to 37.2% in the non-COSAN zone) (see Table 2).

### Descriptive analysis of non-use of coronavirus disease 2019 testing among women

**FIGURE 1:**
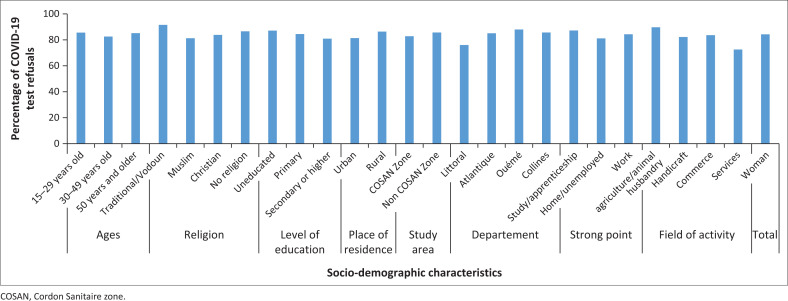
Distribution (%) of respondents reluctant to undergo COVID-19 screening by socio-demographic characteristics.

Figure 1 shows that overall an overwhelming majority (84.2%) of respondents said they had not been tested for COVID-19. The distribution according to socio-demographic characteristics indicates an aversion significantly influenced by: religion, age, area of intervention, level of education, department, activity of women. According to the religion, Vodoun faithful (91.5%) are the most likely to have an aversion to the COVID-19 screening test. They are followed by those ‘without religion’ (86.5%), Christians (83.8%) and Muslims (81.3%). With regards to age, young people (85.6%) and those aged 50 years and over (85.2%) are more resistant to it than those aged 30 years to 49 years (82.5%) (see Figure1).

The non-use of the screening test is inversely proportional to the level of education (uneducated: 87.1%; primary: 84.4%; secondary and above: 80.9%) and more preponderant in rural areas (86.3% compared to 81.3% in urban areas). It is also more dominant in the departments of Ouémé (87.9%), Collines (85.6%) and Atlantique (85.1%) compared to Littoral (76.0%). Depending on the field of activity, women working in the agricultural (89.7%) and commercial (83.6%) sectors are more resistant to the screening test compared to those employed in crafts (82.1%) and other services (72.6%) (see Figure 1).

### Explanatory analysis of the non-use of COVID-19 screening tests

Table 3 shows that the explanatory factors for the non-use of the COVID-19 screening test are: age, religion, level of education, main activity, field of activity and socio-professional category.

**TABLE 3:** Net effect of explanatory variables on non-use of coronavirus disease 2019 screening tests.

Variables and modalities	*B*	s.e.	Wald	*df*	*p*	Exp(B)
**Age (years)**						
15–29	-	-	-	-	-	1
30–49	-0.35	0.11	10.88	1	0.00	0.71
50 years and older	-0.18	0.15	1.40	1	0.24	0.84
**Religion**						
Traditional/Vodoun	-	-	-	-	-	1
Muslim	-0.70	0.21	11.30	1	0.00	0.50
Christian	-0.55	0.18	9.04	1	0.00	0.58
No religion	-0.51	0.29	2.97	1	0.08	0.60
**Matrimonial situation**						
Single	-	-	-	-	-	1
In union	0.19	0.15	1.56	1	0.21	1.21
Separated, divorced and widowed	-0.01	0.20	0.00	1	0.98	0.99
**Level of education**						
Uneducated	-	-	-	-	-	-
Primary	-0.21	0.11	3.71	1	0.05	0.81
Secondary or higher	-0.42	0.11	15.27	1	0.00	0.66
**Place of residence**						
Urban	-	-	-	-	-	1
Rural	0.09	0.10	0.94	1	0.33	1.10
**Study area**						
COSAN Zone	-	-	-	-	-	1
Non-COSAN Zone	-0.07	0.09	0.53	1	0.46	0.93
**Strong point**						
Study/apprenticeship	-	-	-	-	-	1
Home/unemployed	-0.49	0.18	7.68	1	0.01	0.61
Work	-0.85	0.38	5.03	1	0.02	0.43
**Field of activity**						
Agriculture/animal husbandry	-	-	-	-	-	1
Handicraft	-0.48	0.17	7.68	1	0.01	0.62
Commerce	-0.32	0.14	5.04	1	0.02	0.73
Services	-0.49	0.25	3.95	1	0.05	0.61
**Socio-professional category**						
Executive	-	-	-	-	-	1
Worker	0.42	0.34	1.56	1	0.21	1.53
Caregiver	1.20	0.39	9.53	1	0.00	3.31
Independent	0.87	0.32	7.22	1	0.01	2.40
Constant	2.72	0.23	134.99	1	0.00	15.26

*Source*: UAC. Etude sur les effets de la COVID-19 chez les femmes du secteur non formel au Sud et Centre Bénin. University of Abomey-Calavi; 2023

B, regression coefficient; s.e., standard error; *df*, degree of freedom; Exp(B), odd ratio; COSAN, Cordon Sanitaire zone.

Compared to women aged 15–29 years, those aged 30–49 years are 0.7 times less likely not to have a COVID-19 screening test. However, there is no significant difference between respondents aged 15–19 years and those aged 50–64 years in terms of COVID-19 testing.

As far as religion is concerned, compared to followers of traditional religions, Muslim and Christian women are 0.5 and 0.6 times less likely not to have a COVID-19 screening test, respectively. Women in high school or higher are 0.7 times less likely to miss a COVID-19 test compared to those without education. Compared to pupils, students and apprentices, housewives and those who are unemployed are 0.6 times less likely not to take a COVID-19 test (see Table 3).

Compared to women farmers, women craft-workers and those working in the service sector are 0.6 times less likely not to use the COVID-19 test. This risk is 0.7 for women traders. Self-employed women and family workers are respectively 2.4 and 3.2 times more likely not to use the COVID-19 screening test compared to their counterparts in managerial position or workers (see Table 3).

## Discussion

The analysis of the factors of non-use of COVID-19 screening tests highlights the multiplicity and complexity of sources of information on knowledge of the disease and the measures put in place for its control. A recent analysis by the WHO^15^ indicated that incorrect interpretation of health information, which is more common in the event of an outbreak or disaster, negatively influences the mental health of individuals and increases reluctance to respond to measures (barrier measures, testing, vaccination, etc.) and can delay the provision of healthcare. The impact of misinformation on social media includes negative effects such as ‘increased misinterpretation of scientific knowledge, polarisation of opinion, escalation of fear and panic, or less frequent access to health services’.^15^ The quality of information and the speed at which new information is disseminated have a social and health impact. The results obtained, revealed the existence of several factors (individual and contextual) explaining the aversion to COVID-19 testing among women in the informal sector in the south and centre of Benin. Some factors are related to the public health policies, particularly health provision while others are located in the community. Furthermore, some factors are transnational, thus going beyond the organisation of the response at the national level. In all, the majority of women surveyed (84.2%) said they had an aversion to the COVID-19 screening test. This level of aversion is lower than the 94.8% values reported in the United States^3^ and 89.3% in Lagos and Ondo states in Nigeria.^16^ It is, however, much higher than 37.5% reported in Brazzaville in Congo^13^ and 48.9% in Abidjan, Côte d’Ivoire.^17^.

In Benin, except for travellers’ cases, COVID-19 testing was generally voluntary.^18^ This situation is somewhat part of the effort of governments to quickly identify cases and prevent the spread of the disease. The analysis of the profile of people who were not in favour of the screening test indicated that socio-demographic variables (age, level of education, study area and religion) influenced the test. These results are similar to those obtained in Brazzaville, Congo^9^ and Ontario, Canada.^3^

It is noted that the proportion of people who are not favourable to the test is relatively age-oriented, with a higher risk among women under 30 years of age (cases observed in Brazzaville and in Lagos and Ondo states in Nigeria) and inversely with the level of education. It is also higher in rural areas and in the buffer zone (non-COSAN). The result pertaining to age could be explained by the fact that from the beginning of the pandemic, the information conveyed labelled older people with a clinical history as the most at risk of COVID-19, a situation likely to make youngsters feel less responsible. With regard to education, it is noted that a high level of education favoured good knowledge, partly because of more exposure to information (including the infodemic), which could lead to mistrust and not discouragement of the most educated people to take the test. Religious affiliation also influences the non-use of COVID-19 screening tests, with a lower risk for Muslim and Catholic women who have, to a certain extent, complied with state prescriptions for responding to COVID-19 in Benin.

Religious activities correlated with the spread of the virus, especially during the early days of the pandemic; therefore, religious denominations that defied COVID-19 health guidelines were endangering people far beyond the boundaries of the congregation.^19^ Carrying out an income-generating activity reduced the risk of non-use of COVID-19 screening tests among women in central and southern Benin. This situation has already been observed in Ontario, Canada^3^ and in the northwest Ethiopia where unemployed people were less likely to be tested for COVID-19.^20^ Similar discoveries were also made in Nigeria and China.^16^ Even though testing was officially free, some populations still had difficulty meeting the costs of transportation to the testing site.

As far as the field of activity is concerned, compared to women farmers, women craft-workers and women working in the service sector are 0.6 times less likely not to use the COVID-19 screening test. This risk is 0.7 for women traders. Women employed in the agricultural sector most often live in rural areas where health services are generally, poorly provided and of lower quality to meet their needs. To these shortcomings, we must add the lack of good quality information which has led farmers to adopt behaviours contrary to the instructions of the public authorities in the context of COVID-19 in Ethiopia,^21,22^ Bangladesh^23^ and Nigeria.^24^

In general, the context in addition to the poor living conditions, often leads people to abandon public health services, favouring other types of recourse such as self-medication, endogenous medicine, prayers, among others. At the advent of COVID-19, the wait-and-see attitude of decision-makers in some places as well as the infodemic had contributed to disrupting the public initiatives developed as part of the response, so that each citizen was left to their fate, at least to the numinous (‘God punishes to bring order to the world’) to ensure their ‘self-defence’. A disease whose clinical signs are confused with general signs and polymorphic and non-specific respiratory signs and which is already causing a lot of controversy among medical staff.^25^ The generalised phobia induced by the advent of COVID-19 has contributed to the denial of the disease in some communities (‘a disease that scares even white people?’) and has made health facilities (except for caregivers), inhospitable environments to be avoided at all costs so as not to take additional risks of contamination. Distrust has become widespread in the face of a disease that conjures up polysemous and sometimes contradictory social representations, as some of its facets seemed unknown until now, while others were likely to corroborate a geopolitical conspiracy thesis. The aversion to the screening test in these conditions refers to the idea that ‘between two evils, we should abstain or choose the lesser’. Fear of the test result, denial of the disease, negative perceptions of COVID-19 due in part to the infodemic, fear of a positive result that could be considered a fatal outcome and a lack of trust in the health system were also factors that underlie the rejection of the screening test. This is consistent with a study done in Senegal, which found challenges related to stigma and discrimination of infected people, as well as a lack of empathy from care teams and widespread ostracism.^2^ In the context of the fight against human immunodeficiency viruses (HIV) and/or acquired immunodeficiency syndrome (AIDS), several studies had confirmed that the fear of stigmatisation and discrimination led to an attitude of distrust towards HIV and/or AIDS screening.^26,27^

Female managers were at a lower risk than manual workers, family workers and self-employed workers of not taking a COVID-19 screening test. Indeed, access to certain public service structures, banks, international organisations, among others, was conditional on the presentation of a negative result of the COVID-19 test and or a record proving that the anti-COVID-19 vaccination has been done. Failure to comply with these measures could constitute a risk to the maintenance of their jobs or businesses for some women in managerial position or users of public services. The results we have just presented do not indicate any regional differences, in the sense that study areas and place of residence are not significant, and the same is true of marital status. This result could be explained by the fact that the COVID-19 test was made available throughout Benin, in both urban and rural areas.

All of these results also reveal issues of equity and inequality among the main challenges affecting the health of respondents. These issues are global and widely documented by scientific research in public health interventions, particularly in the context of the fight against epidemics. Indeed, recent research works have highlighted Social Inequalities in Health (SIH) as one of the characteristics widely shared in developing countries.^28^ Social Inequalities in Health are considered to be health gaps (mortality, morbidity) systematically linked to gender, socio-professional categories or territories, among others. They can be explained by social, political and cultural determinants resulting from variables such as age, gender, socio-professional category, socio-economic and environmental conditions.^29^ Women’s aversion to COVID-19 testing can be considered a facet of SIH and therefore preventable insofar as it is not only a matter of biology, but of socially constructed determinants. Sometimes inadequate health policies amplify rather than reduce them, especially when the principle of ‘proportionate universalism’ is not integrated into the formulation of public health interventions. In terms of public policy implications of the response to COVID-19, it is not a question of systematically rejecting Western models of health deployed in Africa but, as some studies have indicated, of deconstructing them in order to recontextualise them by integrating locally anchored complements adapted to African systems of representation that are anchored in the local knowledge and collective imagination of the communities that are potential beneficiaries of health interventions.^30^ Considering the unexpected and perverse effects of public health policies – little known to health experts and the voice of ‘contextual’ or local experts can contribute to improving the health of communities.^31^

## Conclusion

The present analyses aimed to study the explanatory factors of the aversion to COVID-19 testing among women in the informal sector in Benin. The results indicate that most respondents were opposed to testing. Young age, level of education, remote areas and endogenous religions were significantly associated with this situation. The fear of testing positive and having to face consequences such as stigma, discrimination and ostracism that would lead to ‘certain death’ were a real obstacle to the promotion of COVID-19 testing.

The insufficient consideration of local contexts or the unexpected effects of public policies in the mechanisms for mobilising populations around health emergencies, the infodemic and SIH have contributed significantly to this aversion. It is important to respect in addition to social norms, volunteerism and motivation at the level of the individual eligible for screening and to ensure that confidentiality, persuasion, good information and good counselling at the screening level are respected by providers. Constant improvement of knowledge about COVID-19 from a holistic perspective, particularly through raising awareness, could improve the acceptability of the screening test.

In terms of implications for public policy, these analyses confirm the complexity of health issues as a political, economic and social matter, the responsibility for which lies not only with public authorities but also with individuals, families and grassroot communities. Likewise, the analysis supports the work of M. De Koninck^32^ that understanding the SIH experienced by populations requires public health approaches based on in-depth knowledge of all the individual, collective and structural factors that influence their health at a population level.

### Limitations of the study

This is a behavioural survey that, in the absence of other evidence, is limited by selection biases (sampling) and the effect (or gap) of memory in respondents. As the sample is representative for the study area, the results cannot be generalised to the whole of Benin, and comparisons must take account of the geographical coverage of the investigation. Its transversal (and therefore, non-diachronic) nature also makes it impossible to follow the trend of situations observed (or related) over time to distinguish those that are stable in a continuum from those that are ephemeral or cyclical. It should also be noted that the pseudo-R-two associated with the model varies from 0.023 to 0.040, indicating that there are other explanatory variables that have not been considered in this study. Finally, there is a lack of qualitative data that could help explain the belief systems that influence COVID-19 screening behaviour.
